# From shield to spear: Charge-reversible nanocarriers in overcoming cancer therapy barriers

**DOI:** 10.3762/bjnano.17.10

**Published:** 2026-01-14

**Authors:** Madhuri Yeduvaka, Pooja Mittal, Ameer Boyalakuntla, Usman Bee Shaik, Himanshu Sharma, Thakur Gurjeet Singh, Siva Nageswara Rao Gajula, Lakshmi Vineela Nalla

**Affiliations:** 1 Department of Pharmacology, GITAM School of Pharmacy, GITAM (Deemed to be University), Rushikonda, Visakhapatnam, Andhra Pradesh, India; 2 GITAM School of Pharmacy, GITAM University, Rudraram, Patancheru, Sangareddy District, Hyderabad, India; 3 GITAM School of Pharmacy, GITAM (Deemed to be University), Visakhapatnam, Andhra Pradesh, India; 4 Chitkara College of Pharmacy, Chitkara University, Rajpura, Punjab-140401, India; 5 Centre for Research Impact & Outcome, Chitkara College of Pharmacy, Rajpura- 140401, Punjab, India; 6 Department of Pharmaceutical Analysis, GITAM School of Pharmacy, GITAM (Deemed to be University), Visakhapatnam, Andhra Pradesh, India

**Keywords:** cancer, charge reversible nanocarriers, nanocarriers, targeted therapy, tumour microenvironment

## Abstract

Cancer remains a significant global health burden, responsible for 16.8% of all deaths and 30.3% of premature mortality due to noncommunicable diseases, and continues to be one of the leading causes of death worldwide despite medical progress. Conventional treatment methods such as surgery, chemotherapy, and radiotherapy often face challenges such as systemic toxicity, drug resistance, and poor tumour selectivity. In response to these limitations, nanotechnology-based drug delivery systems have gained prominence for enhancing solubility, improving molecular stability, enabling controlled drug release, and prolonging systemic circulation, offering superior therapeutic outcomes over traditional approaches. Among these innovations, charge-reversible nanocarriers have attracted considerable attention due to their ability to overcome physiological and pathological barriers in the tumour microenvironment (TME) by altering their surface charge in response to specific stimuli, which enhances drug targeting while reducing off-target effects. These carriers leverage triggers such as changes in pH, enzymatic activity, redox conditions, temperature, light, ultrasound, X-rays, and magnetic fields to enable intelligent and controlled release of therapeutics. This review examines the crucial role of surface charge in cellular uptake and intracellular transport, highlighting recent advances that demonstrate improved targeting, reduced systemic toxicity, enhanced cellular internalisation, and the potential for integrated approaches, including combination therapies and theranostics. Despite these promising developments, challenges related to nanocarrier stability, safety, manufacturing scalability, and regulatory approval still impede clinical translation. Nevertheless, emerging trends in nanocarrier design, the advancement of personalised medicine, and integration with therapies (e.g., immunotherapy) underscore the transformative potential of charge-reversible nanocarriers in revolutionising cancer treatment and improving patient outcomes.

## Review

### 1 Introduction

Cancer remains a foremost global health challenge, characterized by uncontrolled cellular proliferation and the ability to invade and metastasize to distant sites. Unlike normal cells, cancer cells bypass regulatory mechanisms to form tumours and spread via lymphatic or circulatory systems, such as malignant breast epithelial cells metastasizing to axillary lymph nodes [1]. Leukaemias and other haematological malignancies spread differently, affecting the bone marrow, lymph nodes, and the blood [2]. According to the latest GLOBOCAN and World Health Organization data, cancer ranks as the leading cause of death among individuals aged 30 to 69 in 177 countries, accounting for 16.8% of all deaths globally and 30.3% of premature mortality from noncommunicable diseases [3]. Traditional cancer treatments, such as chemotherapy, hormonal therapy for hormone-sensitive cancers, and radiation therapy, primarily aim to eliminate rapidly dividing cancer cells. However, these conventional approaches often face limitations in specificity and long-term efficacy [4–6]. In recent years, significant advancements have transformed the therapeutic landscape with the introduction of gene therapy, stem cell therapy, targeted therapy, and immunotherapy modalities that enable more precise and personalized cancer management [7]. In parallel, complementary strategies such as photodynamic therapy and hyperthermia further enhance treatment effectiveness, collectively reflecting the ongoing evolution of cancer therapeutics [8]. Despite their efficacy, conventional therapies often cause severe side effects. Chemotherapy induces anaemia and immunosuppression, radiation triggers fatigue and psychological strain, surgery carries risks of organ damage, and hormone therapy disrupts endocrine balance, highlighting the pressing need for innovative solutions. An example would be smart nanocarrier drug delivery systems that enhance targeting precision and mitigate adverse effects [9–10].

**Figure 1:**
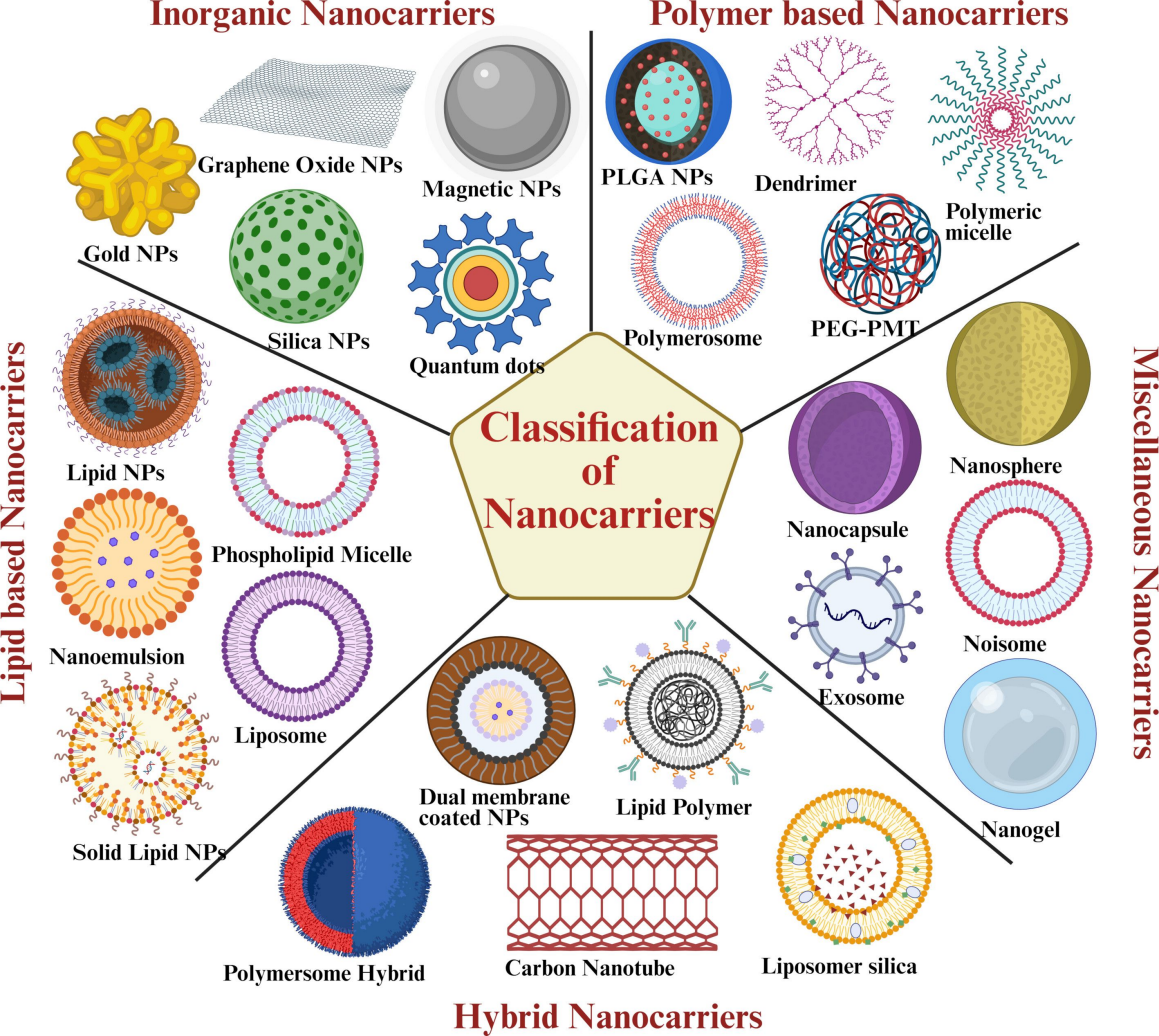
Classification of nanocarriers. This schematic depicts the main classes of nanocarriers for drug delivery systems. Figure 1 was created in BioRender. Yeduvaka, M. (2025) https://BioRender.com/txbz21s. This content is not subject to CC BY 4.0.

Nanotechnology-based drug delivery systems have revolutionised cancer treatment by improving drug solubility, stability, and biodistribution while protecting fragile biomolecules such as proteins and nucleic acids [11]. Through targeted and sustained release, these systems enhance therapeutic efficacy, prolong circulation, and reduce systemic toxicity compared to conventional formulations [12–13]. As illustrated in Figure 1, nanocarriers encompass diverse types including polymeric nanoparticles, liposomes, micelles, dendrimers, lipid-based carriers, carbon-based nanomaterials, and gold nanoparticles.

They exhibit versatile structures (1–100 nm) with diverse morphologies (e.g., spherical, tubular, or conical shapes [14]). With advancements in nanocarrier-based cancer therapy, recent research has increasingly emphasised refining their physicochemical traits, especially surface charge, to boost therapeutic outcomes. A notable development in this context is the emergence of charge-reversible nanoscale drug delivery systems (CR-NDDSs) [15]. These systems can switch their surface charge in response to tumour microenvironment (TME) triggers such as pH changes, redox states, or enzymatic activity, enhancing drug stability, facilitating cellular uptake, and enabling targeted drug release. This responsive functionality gives CR-NDDSs a distinct edge over conventional nanocarriers, representing a significant leap toward more precise and efficient cancer treatments [16]. This review aims to present an in-depth analysis of charge-reversible nanocarriers (CRNs) in cancer treatment, emphasising their underlying mechanisms, benefits, and therapeutic value. It also explores existing challenges and prospects to advance their translation into clinical cancer treatments.

### 2 Concept of charge-reversible nanocarriers

**Figure 2:**
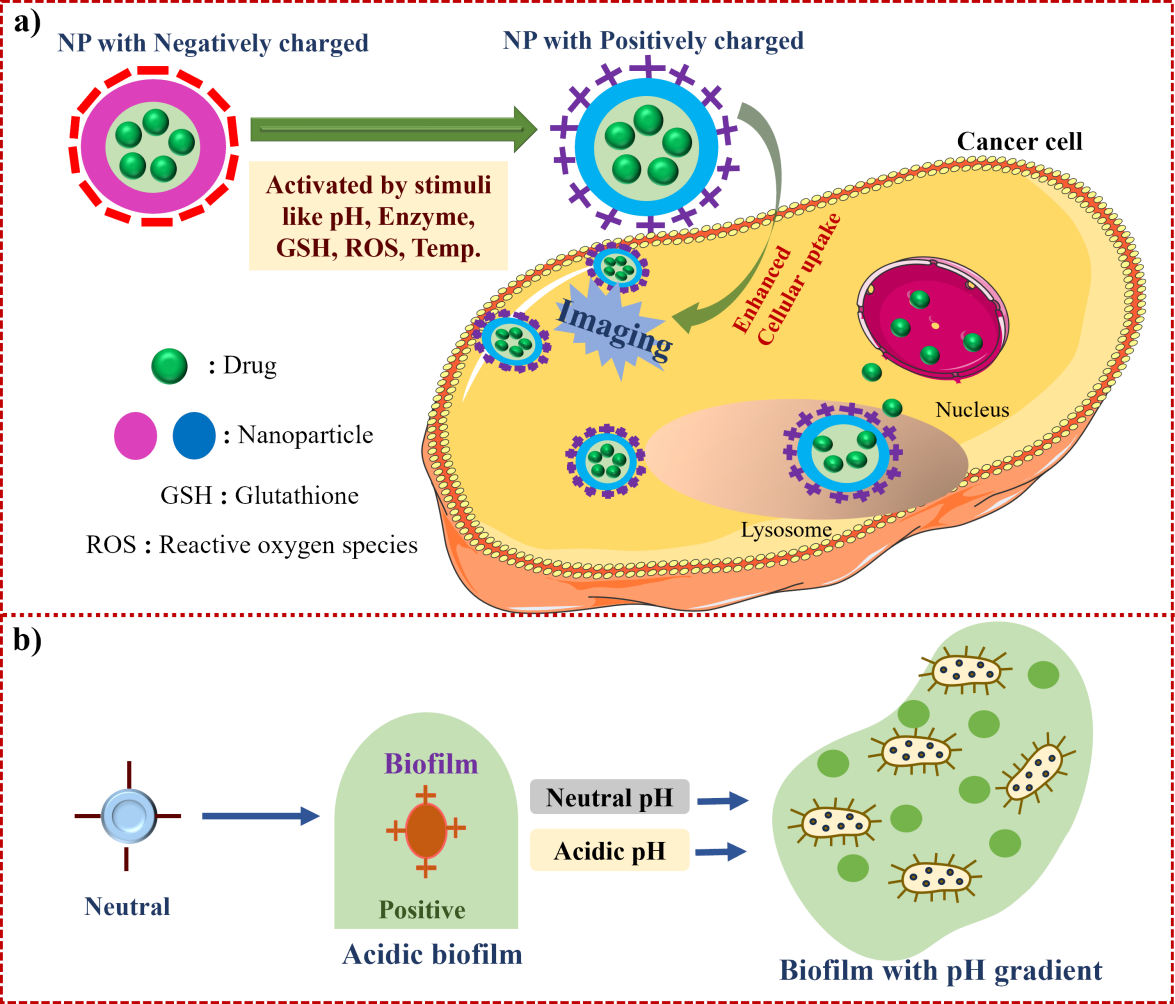
Schematic representation of charge-reversible nanocarrier system for tumour therapy. a) In general mechanism of CRNs activated by various stimuli for targeted therapy, b) charge reversal of nanocarriers exhibiting responsiveness to the acidic environment in biofilms. The images of EMPTY CELL, NUCLEUS and PROTEIN were provided by Servier Medical Art (https://smart.servier.com/), licensed under CC BY 4.0 (https://creativecommons.org/licenses/by/4.0/).

Surface charge plays a vital role in the efficiency and functionality of nanocarriers used for drug delivery [13]. Among various physicochemical parameters, the surface charge is essential for determining nanocarrier interactions with biological membranes, cellular uptake, and biodistribution [17]. With a positive charge, the nanocarrier tends to be absorbed by high plasma proteins and cleared faster from the bloodstream. In contrast, those with neutral or negative charge exhibit longer circulation times, reduced immune clearance, and improved therapeutic efficacy. Further, positively charged nanocarriers exhibit enhanced cellular uptake due to their electrostatic interaction with negatively charged cell membranes; however, they may also induce cytotoxicity and rapid clearance by the mononuclear phagocyte system [18]. Moreover, surface charge influences aggregation behaviour, colloidal stability, and protein corona formation, directly impacting the therapeutic efficacy of nanocarriers. Optimising surface charge is essential for enhancing the therapeutic efficacy and safety profiles of nanocarriers in clinical applications [15]. The functional mechanism of CRNs (Figure 2) is designed to enhance the selectivity and efficacy of anticancer drug delivery systems, along with their behaviour in response to the acidic environment of biofilms within the TME, where the nanoparticles are activated by pH changes and demonstrate their potential for site-specific drug delivery [19].

This strategy also enables controlled drug release, as the pH-sensitive charge reversal triggers site-specific drug unloading in acidic tumour microenvironments or intracellular compartments like endosomes and lysosomes [20]. Bobrin et al. studied that a PEI-based polymeric nanocarrier demonstrated charge reversal from negative (pH 7.4) to positive (pH 6.8) under tumour pH. This transformation facilitated tumour cell uptake and site-specific unloading of siRNA within lysosomes via protonation-induced release [21]. Similarly, a study presented a polymer nanocarrier with acid-triggered charge reversal achieving >60% drug release within 48 h at lysosomal pH 5.0, compared to less than 10% release at physiological pH 7.4. The system used tethered imidazole groups for protonation-driven charge inversion, ensuring precise intracellular payload unloading [22]. Furthermore, the neutral charge state during systemic circulation helps to reduce cytotoxicity by minimising nonspecific protein adsorption and immune system activation. A study by Yuan et al. showed that zwitterionic and neutral nanoparticles possess highly hydrated, charge-balanced surfaces that minimize serum protein adsorption, complement activation, and cytokine release (IL-6, TNF-α). In murine models, these particles exhibited reduced systemic inflammation and enhanced circulation stability [23]. Neutral PEG or hydroxyl-modified nanoparticles demonstrated significantly lower protein binding, opsonization, and phagocytic uptake compared to their charged counterparts, thereby reducing immune clearance and cytotoxicity [24].

**Figure 3:**
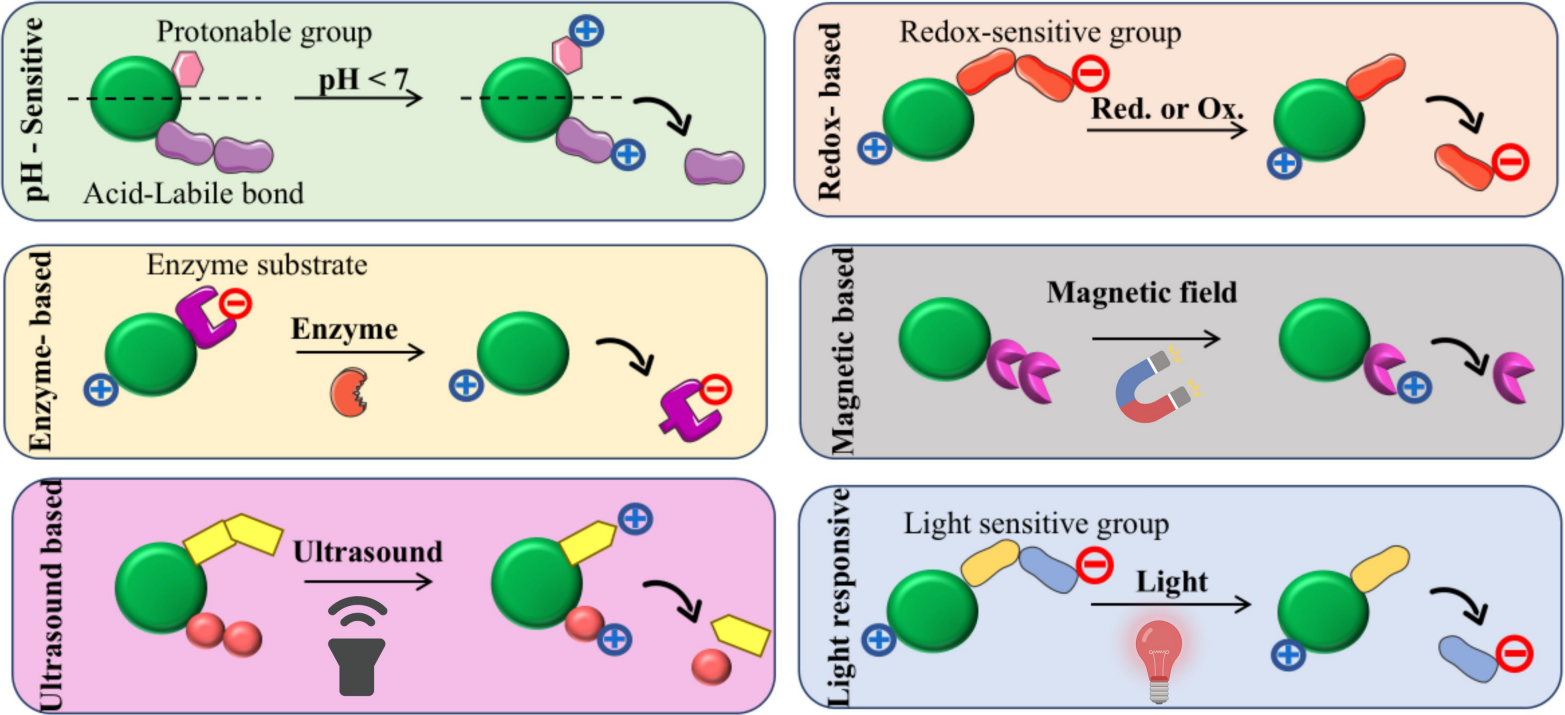
Illustrative mechanisms of various charge-reversible nanocarrier (CRN) types. Figure 3 was created in BioRender. Yeduvaka, M. (2025) https://BioRender.com/2d2dp36. This content is not subject to CC BY 4.0.

Overall, these findings demonstrate that surface charge modulation through pH-sensitive or neutral/zwitterionic designs enhances therapeutic precision, circulation stability, and biocompatibility by enabling controlled drug release while minimising immune recognition and cytotoxicity. CRNs exhibit diverse mechanisms, applications, and advantages that enhance their performance in targeted and controlled drug delivery (Table 1). The detailed mechanism of CRNs in response to various environmental stimuli is described in Figure 3.

**Table 1:** Types of charge-reversible nanocarriers, their mechanisms, applications, and advantages.

Type of charge-reversible nanocarrier	Mechanism of charge reversal	Application in drug delivery	Advantages	Examples	Ref.

pH-sensitive	protonation/ deprotonation in acidic tumour environment (pH 6.5–6.8)	targeted drug release in acidic tumour sites and intracellular compartments	enhanced tumour penetration, improved cellular uptake, and minimised off-target effects	doxorubicin-loaded pH-sensitive liposomes; polymeric nanoparticles with pH-responsive bonds	[25–27]
enzyme- responsive	enzyme-mediated cleavage of functional groups or linkers	site-specific drug release where enzymes (e.g., MMPs) are overexpressed	high specificity, tumour-selective activation, improved efficacy	MMP-responsive peptide-modified nanoparticles	[28–30]
redox-sensitive	disulfide bond cleavage triggered by high glutathione (GSH) levels	intracellular drug release in reductive tumour environments	increased drug concentration in tumour cells, reduced systemic toxicity	GSH-responsive disulfide-linked polymeric micelles; DTPA-DOX nanoparticles	[31–33]
light- responsive	structural change or ROS generation upon light exposure (UV–NIR)	on-demand drug release via external light	spatial/temporal control, selective tumour targeting, phototherapy compatibility	spiropyran-based nanocarriers; UV-sensitive nanocarriers	[34–36]
ultrasound- responsive	cavitation or thermal effects trigger reversible interactions	enhanced drug penetration through deep tissues	noninvasive targeting, controlled release, and high biocompatibility	ultrasound-triggered liposomes or microbubbles	[37–38]
X-ray- responsive	ROS generation and bond cleavage under X-ray exposure	drug release during radiotherapy for synergy	enhanced radiotherapy, reduced drug dosage, dual therapy	gold nanoparticle-based radiosensitizers	[39–41]
magnetic- responsive	localised hyperthermia via alternating magnetic field	magnetically guided, localised drug release	noninvasive precision therapy, MRI compatibility, controlled targeting	SPIONs-based magnetic liposomes	[42–44]

#### 2.1 pH-responsive charge-reversible nanocarriers

When exposed to the acidic cancer microenvironment (pH 6.5–6.8), CRNs undergo a pH-sensitive change in their surface charge [4]. The breakdown of adenosine triphosphate to release protons in the cancer tissue, along with aerobic glycolysis, which generates lactic acid, results in a decrease in the pH of the TME, which is a widely observed phenomenon [22]. Typically, the pH of most solid tumours ranges between 5.7 and 7.8, differing significantly from the physiological blood pH of 7.4 [23]. These nanocarriers typically possess pH-sensitive chemical bonds or functional groups that undergo protonation or deprotonation, leading to a charge switch from negative to positive. This charge reversal enhances cellular uptake by facilitating better interaction with the negatively charged cell membrane, improving drug delivery efficiency [45]. For example, nanoparticles engineered with pH-responsive polymers facilitate the controlled release of therapeutic agents specifically within the acidic tumour microenvironment, thereby improving treatment efficacy. A notable study by Liu Y et al. described polymeric micellar nanoparticles incorporating hydrazone bonds within their core–shell structure; these hydrazone linkages are selectively cleaved under acidic conditions, such as those found in endosomes and lysosomes. Upon bond cleavage, the nanoparticles undergo a surface charge shift by exposing cationic groups, which significantly enhances cellular adsorption and uptake. This charge-switching mechanism, exemplified by PEG-*b*-poly(ʟ-lysine)-hydrazone- doxorubicin micelles from the cited study, enables targeted drug delivery with improved intracellular release and reduced systemic toxicity, highlighting the therapeutic advantage of pH-sensitive hydrazone bonds in nanocarrier design [46]. Another work involved polymers modified with weak acidic groups (e.g., carboxylic acids or phosphates). Under neutral pH, the surface was negatively charged, but in acidic tumour tissues or endosomes, protonation caused a charge reversal to positive, facilitating cell binding and internalisation, which improved drug accumulation within tumour cells [21]. These scientific findings exemplify the use of pH-sensitive bonds and functional groups that enable charge reversal, a strategy that significantly improves cellular interaction and drug delivery efficiency in tumour environments. Additionally, it should be noted that, unlike pH-responsive charge reversal, which is reversible due to protonation and deprotonation processes, other stimuli, such as enzymatic cleavage, redox reactions, or magnetic heating induce irreversible charge changes since they involve permanent chemical or structural modifications to the nanocarrier surface.

#### 2.2 Enzyme-responsive nanocarriers

Enzymes are essential components that manage cellular function and various bodily processes. Certain enzymes, such as matrix metalloproteinases (MMPs), hyaluronidases (HAases), γ-glutamyltransferases (GGTs), aminopeptidases (APNs), esterases, and others, are found more regularly in cancer cells [32,47–49]. By encapsulating specific enzyme substrates in nanocarriers, novel drug delivery systems may respond to overexpressed enzymes both inside and outside cells. CRNs that are enzyme-responsive specifically utilise the enzyme presence in the TME to trigger drug release. A study by Liu et al. developed nanomicelles that respond to cathepsin B overexpressed in tumours, undergoing enzymatic cleavage of peptide bonds which causes nanocarrier destabilization, charge reversal, and size reduction. These transformations triggered drug release within the tumour tissue and improved nuclear targeting, optimizing therapeutic efficacy [50]. Thereafter, a study by Lin et al. developed MMP-2-responsive PEG-coated nanocarriers that, upon peptide cleavage, underwent PEG detachment, charge reversal, and size reduction, enhancing cellular uptake and tumour-specific drug release. This system effectively inhibited tumour growth with minimal systemic toxicity in mice [51]. These studies demonstrate how enzyme substrates embedded in nanocarriers enable the selective cleavage, charge reversal, and targeted delivery of drugs in response to cancer-associated enzymes.

#### 2.3 Redox-sensitive nanocarriers

Redox-sensitive nanocarriers exploit the distinct intracellular environment of tumour cells, characterized by elevated levels of GSH and reactive oxygen species (ROS), to trigger on-demand drug release. These nanocarriers typically incorporate redox-sensitive chemical bonds, such as disulfide linkages, which remain stable in blood circulation but undergo cleavage in the reductive tumour microenvironment, leading to structural transformation and payload discharge. By taking advantage of the high GSH concentration and acidic extracellular pH of the tumour, these systems can achieve precise site-specific drug release and improved therapeutic efficacy while minimizing systemic toxicity [52]. For example, a nanocarrier constructed by conjugating 2,3-dimethylmaleic anhydride (DMA) and 3,3-dithiopropionic acid-modified doxorubicin (DTPA-DOX) onto poly(ethylene glycol)-*b*-poly(ʟ-lysine) (PEG-*b*-PLL) encapsulates triptolide in its hydrophobic core. Under acidic tumour extracellular pH, the DMA group triggers a charge reversal, improving cellular uptake, whereas the high intracellular GSH levels cleave the disulfide bond in DTPA-DOX, releasing the drug payload specifically inside tumour cells. This dual redox and pH-sensitive strategy ensures controlled and efficient delivery of multiple drugs in response to the biochemical cues of the tumour microenvironment, enhancing anticancer activity with reduced off-target effects [53].

#### 2.4 Light-responsive nanocarriers

Light-sensitive nanocarriers represent an innovative approach to regulated drug delivery, utilizing photosensitive materials such as graphene, azobenzene, and gold nanorods [54]. Upon exposure to UV–vis or near-infrared (NIR) light, these materials undergo structural changes or generate ROS, triggering controlled release of their therapeutic cargo. This precise spatiotemporal control over drug release and therapeutic activity enhances treatment efficacy and minimises off-target effects, thus seamlessly complementing other stimuli-responsive delivery systems discussed earlier.

Earlier, a study by Hu et al. demonstrated that nanocarriers functionalized with photoisomerizable azobenzene groups, upon UV–vis or NIR light exposure, azobenzene undergoes reversible trans–cis isomerisation, inducing structural changes that regulate cargo release [55]. Additionally, Choi et al. demonstrated that nanocarriers incorporating graphene oxide (GO) loaded with photosensitizers generate ROS upon NIR irradiation, enabling effective tumour photodynamic therapy. The study highlights the excellent light absorption and ROS generation capacity of the material, allowing targeted tumour cell damage while sparing healthy tissues [56]. Similarly, a study reports thermo-responsive gold nanorod vesicles (USGRV-17-AAG) integrate NIR-II photothermal therapy and chemotherapy by encapsulating the HSP90 inhibitor 17-AAG within UCST-type polymer-modified gold nanorods. Upon 1064 nm irradiation, they exhibit 65.1% photothermal conversion efficiency and trigger heat-induced 17-AAG release, achieving 98.86% of tumour growth inhibition in mice [57]. Collectively, these studies validate that light-sensitive nanocarriers incorporating photosensitive materials are capable of controlled drug release and phototherapeutic tumour ablation with high specificity and minimised collateral damage.

#### 2.5 Ultrasound-responsive nanocarriers

Ultrasound-responsive CRN is a novel technique for precise and effective gene or drug delivery. When exposed to ultrasonic treatment, the dynamic coordinating leakages, such as carboxyl–calcium interaction, on which these nanocarriers rely, may be reversibly broken and re-established. For example, a study by Li et al. designed calcium-ion-crosslinked sodium-alginate-coated mesoporous silica nanoparticles (MSNs) for ultrasound-triggered drug delivery. High- or low-intensity focused ultrasound (HIFU/LIFU) induced reversible disruption of carboxyl–calcium bonds, enabling precise, on-demand release [58]. This confirms that ultrasound-responsive CRNs employing calcium-ion-crosslinked sodium-alginate coatings on mesoporous silica nanoparticles offer reversible, on-demand, and biocompatible drug release options. Such systems harness ultrasound-induced cavitation to disrupt and reform ionic bonds, showing considerable promise for cancer therapy and other clinical applications.

#### 2.6 X-ray-responsive nanocarriers

These systems offer innovative mechanisms for targeted drug delivery systems and enhanced therapeutic efficiency. These nanoparticles are designed to release therapeutic agents upon exposure to X-rays, which can generate ROS [59] and activate drug release mechanisms. For instance, nitroimidazole-ligated gold nanoparticles release nitrate, a precursor for nitric oxide, when irradiated with clinically relevant X-rays. This release sensitises hypoxic cancer cells to radiation therapy by generating reactive oxygen and nitrogen species, thereby improving treatment outcomes [60]. Additionally, an X-ray-activated nanoscale platform can produce significant quantities of ROS-enhancing PDT effects in cancer treatment by conjugating photosensitizers to these nanoparticles; the efficiency of ROS generation increases under X-ray radiation compared to that under conventional methods. These dual-functionality CRNs improved drug delivery precision and enhanced the overall effectiveness of radiotherapy [61]. A study by Liu et al. developed the nanoscale coordination polymer Hf-nIm@PEG (HNP), which integrates hafnium ions (Hf^4+^) with 2-nitroimidazole and a PEG-modified lipid shell, enabling multifunctional X-ray-responsive therapy. Upon low-dose of X-ray irradiation, Hf^4+^ deposits radiation energy to induce DNA damage while 2-nitroimidazole releases NO to block DNA repair, relieve hypoxia, and produce reactive nitrogen species (RNS) that trigger apoptosis. Moreover, Hf^4+^ activates the cGAS–STING immune pathway, enhancing antitumour immunity and achieving synergistic radio-immunotherapy against cancer [62].

#### 2.7 Magnetic-responsive nanocarriers

Magnetic-responsive CRNs utilise magnetic fields to enhance drug delivery and therapeutic efficiency [63]. Superparamagnetic iron-oxide nanoparticles (SPIONs), which react to external magnetic fields and enable selective accumulation in tumour areas, are commonly used to create these nanocarriers [64]. These systems follow thermo-sensitive binding strategies that permit targeted drug release (e.g., DOX, geldanamycin) under alternating magnetic fields while preserving systemic safety [65]. Core–shell magnetic nanoparticles (Fe_3_O_4_@P(MEO_2_MA_60_-OEGMA_40_)) combine magnetic hyperthermia with controlled doxorubicin release, achieving localized heating (≈42 °C) under an alternating magnetic field to trigger drug release. They show minimal release at physiological temperature, near-complete release under hyperthermia, and excellent tumour-targeting efficacy with high biocompatibility [66]. Iron oxide nanocubes coated with a thermoresponsive polymer (TR-DOXO) enabled magnetic-hyperthermia-triggered doxorubicin release at temperatures of ≥44 °C, effectively targeting resistant and quiescent cancer stem cells. Combined TR-DOXO and magnetic field treatment achieved complete tumour inhibition in mice, demonstrating strong hyperthermia–chemotherapy synergy [67]. The combination of magnetic targeting, thermal stimuli responsiveness, and charge-reversal mechanisms offers a powerful route to overcome multidrug resistance and enhance cancer treatment precision.

### 3 Role of charge-reversible nanocarriers in cancer therapy

Polymer-based CRNs have shown significant promise in improving cancer treatment by precisely regulating drug activity within the tumour microenvironment. Their capacity to switch surface charge in response to specific biological triggers enhances therapeutic effectiveness [68]. This section highlights the diverse functions of CRNs, such as enhanced targeting, minimised off-target effects, controlled drug release, and co-delivery strategies. Furthermore, their use in theranostics and promoting cellular uptake emphasises their potential in advancing personalised and more efficient cancer therapies. An overview of various CRNs employed in targeted cancer therapy is presented in Table 2, illustrating their key functionality, mechanism of drug release, and surface modification.

**Table 2:** Overview of charge-reversible nanocarriers in targeted cancer therapy.

Nanoparticle type	Targeted cancer therapy	Functional groups involved in charge reversal	Mechanism of drug release	Surface modification	Key results	Ref.

Role of CRNCs: targeted delivery						

pH-activatable supramolecular MI7-β-CD/SA NCs	hepatocellular carcinoma	protonation/ deprotonation of the carboxyl (–COOH/–COO^−^) and imidazole (–NH^+^/neutral) groups on MI7-β-CD/SA nanoparticles	diffusion- based drug release	sodium alginate and imidazolyl- decorated cyclodextrin	- 90% cumulative release of celastrol at pH 5.0 - high apoptosis rate	[69]
pH/hypoxia- responsive COF-based NCs	anticancer drug delivery for solid tumours	protonation of imine (–C=N–) groups under acidic pH causes surface charge to switch	hypoxia reduction of azobenzene to amines	zwitterionic polymer (sulfamide- based)	- surface potential rose from −15.45 mV at pH 8.0 to 12.24 mV at pH 5.4 - charge switched from negative to positive at pH ≈6.5, attributed to protonation of imine groups	[70]
dual pH/redox- sensitive PMAABACy/CS/CS-DMMA NPs	doxorubicin to tumour cells	cleavage of DMMA–amide bonds at acidic pH exposes chitosan amines (–NH_2_), switching surface charge	GSH-triggered degradation of sulfide cross-links in the intracellular tumour environment	dimethylmaleic anhydride-modified chitosan (CS-DMMA)	- PMAABACy cores showed an initial negative zeta potential of −38.2 mV - after adsorption of the cationic CS layer, the potential reversed to +29.3 mV - following the addition of the CS-DMMA polyanion layer, the potential shifted back to negative, measuring −28.4 mV	[71]
mesoporous silica NPs (MSNs-COS-CMC)	cervical carcinoma	the carboxyl (–COOH) and amino groups on carboxymethyl chitosan (CMC) and chitosan oligosaccharide (COS) are responsible for the charge reversal	endocytic uptake and pH-triggered release for enhanced delivery	chitosan oligosaccharide and carboxymethyl chitosan	- higher cytotoxicity at acidic pH (6.5): IC_50_ = 0.6 µg/mL - much lower IC_50_ than at physiological pH (7.4): IC_50_ = 5.8 µg/mL - more potent than free DOX at pH 6.5: free DOX IC_50_ = 2.6 µg/mL	[72]

Role of CRNCs: reduced off-target effects						

GelMA-PEDOT- based NCs	breast cancer and other tumours	the thiophene groups (C–S–C) of the PEDOT backbone and the associated p-toluenesulfonate (–SO_3_^−^) dopant ions undergoing charge reversal	controlled release via electrical or environmental triggers	functionalized with GelMA hydrogel and PEDOT polymer	- improved tumour uptake, reduced off-target toxicity, tuneable release	[73]
mesoporous silica-based NCs (MCM@CS@PEG-APT)	breast cancer	the amino groups on chitosan, which protonate to –NH_3_^+^ under acidic pH	increase DOX uptake and 73% release at pH 5.5 over 10 days	chitosan and polyethylene glycol coating for biocompatibility, with aptamer functionalization	- 99.42% DOX loading - 98% cell uptake - 42.7% co-delivery (DOX + DNA)	[74]
mixed micelles with nimotuzumab (NTZ-DCMMs)	hepatocellular carcinoma	protonation of –NH_2_ to –NH_3_^+^ and deprotonation of –COOH to –COO^−^ under varying pH causes surface charge reversal	reduction-sensitive cleavage of disulfide bonds in PEG-*b*-P(GMA-ss-DOX)	nimotuzumab (anti-EGFR antibody)	- enhanced tumour drug accumulation, inhibited growth, and reduced cardiotoxicity	[75]
mesoporous silica nanoparticles (MSNs)	HER2-positive breast cancer	amine (–NH_2_) and carboxyl (–COOH) groups undergo protonation and deprotonation, driving the reversible charge reversal of the nanocarriers	controlled release via pH-sensitive poly(tannic acid) "gatekeeper"	poly(tannic acid) polymer shell with HER2 antibody conjugation	- efficient tumour inhibition with minimal side effects and low myocardial toxicity - 2.2× higher tumour targeting using pH-responsive nanocarriers - selective drug release: 67.9% at acidic pH vs 8.1% at physiological pH - improved cytotoxicity: IC_50_ = 0.32 µg/mL (nanocarrier) vs 0.42 µg/mL (free drug).	[76]

Role of CRNCs: controlled drug release						

redox/pH dual-responsive HA-SH/CS nanoparticles	breast cancer	amino groups, which are protonated to –NH_3_^+^ in acidic environments	GSH-triggered disulfide bond cleavage and pH-triggered release at acidic intracellular environments	thiol-hyaluronic acid (HA-SH) and chitosan (CS) for charge reversibility and CD44 targeting	- high DOX-loading (45.7 wt %), rapid release (87.8 wt % at pH 4.5, 10 mM GSH), and improved cellular uptake	[77]
pH-responsive charge-reversal polyelectrolyte and integrin αvβ3 mono-antibody	DOX to cancer cells (U87 MG)	citraconic amide (–CONH–C(COO^–^)CH_3_) moieties on PAH-Cit, which hydrolyse to expose protonated amine groups on poly(allylamine)	releasing DOX from GO into the nucleus	functionalized GO with charge-reversal polyelectrolytes and integrin αvβ3 mono- antibodies	- high DOX loading, targeted U87 MG cell uptake via αvβ3, and effective nuclear delivery for enhanced therapy - at physiological pH (pH 7.4), PAH-Cit is positively charged - specifically, at pH 5.0, PAH-Cit reverses to a negatively charged state	[78]
pH-responsive charge-reversal and photo- crosslinkable polymer NPs	DOX with inhibitory effects on tumour cell growth	dimethylamino (–N(CH_3_)_2_) and carboxyl (–COOH/–COO^−^) groups undergo protonation– deprotonation	pH-dependent release and UV-triggered photo-cleavage enable precise DOX control	coumarin- functionalized copolymer with reversible photo-reactions (λ = 365 nm cross-linking, λ = 254 nm cleavage)	- pH ≤ 4: micelles show a positive zeta potential (≈ +19.44 mV) - pH 5.0–7.8: zeta potential decreases and approaches 0 mV - around pH 8.6: zeta potential is near neutral (≈ 0 mV) - pH > 10: nanoparticles exhibit a negative zeta potential (≈ –27.88 mV)	[79]
pH-responsive charge-reversal polymer-coated mesoporous silica NPs	cervical carcinoma	charge reversal occurs via citraconic amide groups, which hydrolyse to primary amine groups under acidic conditions	acidic pH triggers charge reversal, facilitating drug release and endosome escape to ensure nuclear targeting	coating with poly(allylamine)-citraconic anhydride (PAH-cit) and (3-aminopropyl)triethoxysilane (APTES)	- efficient DOX delivery to nuclei, real-time confocal laser scanning microscopy imaging, and effective cancer cell killing with good biocompatibility	[80]

Role of CRNCs: combination therapy (or) co-delivery						

dual-responsive shape- transformable charge-reversible nanomedicine system (DHP@BPP)	breast cancer and lung metastasis	imidazole groups of histidine residues undergo protonation, causing charge reversal	acidic TME protonates histidine, inducing DHP shedding and charge reversal; MMP-2 cleaves BPP to release BBR, and ROS from PPA under 650 nm laser enhances ICD	PEG-modified dPPA for tumour targeting; DHP electrostatically adsorbed onto BPP for charge reversal and extended circulation	- charge reversal in DHP@BPP NPs was quantitatively confirmed by a zeta potential shift from −16.4 to +12.5 mV at pH 6.5 - results in 1.62× deeper tumour penetration and significantly enhanced cellular uptake under acidic, MMP-2-rich conditions	[81]
metal–organic framework (MOF)-based polymer-coated hybrid NPs	breast cancer	charge reversal occurs via hydrolysis of ortho ester groups, exposing amine groups on the inner MOF core	polymer degrades to expose MOF core, enabling multi-drug release, nuclear targeting, and tumour penetration	polymer coating stabilises and masks charge, revealing cationic MOF core in acidic tumours	- surface charge stayed negative (−33 mV) at pH 7.4 - charge switched to positive (+28 mV) at tumour-relevant pH 5.0 within 4 h, indicating polymer degradation and exposure of the cationic MOF core. - MCF-7/ADR cells showed significantly higher uptake at acidic pH, with ≈3.5-fold greater DOX fluorescence at pH 6.5 compared to pH 7.4	[31]
silk sericin-based nanoparticles (MR-SNC)	breast cancer	amino, carboxyl (–COOH), and hydroxyl (–OH) groups in sericin undergo ionization, causing pH-dependent charge reversal	pH-dependent release: maximum at mildly acidic pH (pH 6)	pH-triggered charge reversal boosts cellular uptake and drug release by disrupting sericin- electrostatic interactions	- optimal nanoparticle size (≈127 nm), reduced MCF-7 viability, enhanced uptake at pH 6, and induced DNA damage and apoptosis	[82]
dual-pH responsive DMMA-P-DOX/ LAP polymeric nanoparticles	breast cancer	primary amine groups on e-poly-ʟ-lysine undergo charge reversal via acid-labile β-carboxylic amide cleavage	pH-triggered drug release in endo/ lysosomes disassembles nanoparticles, releasing LAP for a synergistic anti-tumour effect	a dual-pH responsive surface enhances tumour targeting and endosomal escape	- stable circulation, selective tumour accumulation, and significant tumour reduction/complete elimination in the MCF-7 cells	[83]

Role of CRNCs: improved cellular uptake						

dual pH-sensitive charge-reversal poly(β-ʟ-malic acid) (PMLA)-based nanocomplex	anticancer activity	dimethylmaleic anhydride (DMMA) and amino groups undergo hydrolysis, exposing amines and reversing surface charge	at pH 6.8, DMMA hydrolyses, exposing TAT and reversing charge; at acidic pH, micelles release DOX	surface coated with pH-sensitive PEG-DMMA	- at pH 7.4, the nanocomplex maintained a negative surface charge of −16.33 mV while the surface charge reversed to +10.81 mV at pH 6.8	[84]
charge- convertible carbon dots (CDs−Pt(IV)@ PEG-(PAH/DMMA))	anticancer activity	DMMA groups hydrolyse in acidic pH, exposing amino groups that switch surface charge	at pH 6.8, the polymer shifts to positive, enhancing internalisation; reductive cytosol activates cisplatin prodrug	functionalized with PEG-(PAH/DMMA) for pH-responsive charge reversal and enhanced biocompatibility	- prolonged blood circulation enabling effective tumour targeting - enhanced therapeutic efficacy with reduced side effects - pH-responsive surface charge switching: At pH 7.4: zeta potential = −16.15 mV At pH 6.8: zeta potential shifts to +12.01 mV after 4 h at 37 °C	[85]

#### 3.1 Targeted delivery

The charge-reversible NPs encapsulating diagnostic probes and therapeutic drugs result in efficient tumour-targeted delivery [86]. To achieve targeted delivery, charge reversal needs to be precisely controlled within a small pH range [87]. Chen et al. developed pH-activatable charge-reversal supramolecular nanocarriers, named MI_7_-β-CD/SA NPs, which show targeted delivery and controlled release of celastrol in tumour cells, enhancing drug accumulation and therapeutic effects while minimising toxicity to normal cells [69]. Wang et al. developed a pH/hypoxia synergistic nanocarrier technology, which, with the aid of azo and sulfamide-based zwitterions, achieved targeted medication release and deep tumour penetration [70]. Miao et al. designed a pH/reduction-sensitive, charge-reversal PMAABACy/CS/CS-DMMA nanohydrogel, with excellent biodegradability and biocompatibility, which holds strong potential as a doxorubicin drug carrier for targeted nuclear delivery in cancer therapy [71]. The charge-reversal DOX@MSNs-COS-CMC nanocarrier designed by Cui et al. for targeted delivery, demonstrated enhanced uptake and nuclear delivery in HeLa cells, reduced inflammatory cytokines, and improved tumour inhibition over free DOX, showing promise for cervical carcinoma therapy [72]. Cheah et al. developed a charge-responsive CPH material enabling electrically controlled protein release, reducing off-target effects through tuneable, site-specific delivery via GelMA-PEDOT interactions and degradation over 21 days [73]. Esmaeili et al. developed a charge-reversible MCM@CS@PEG-APT (DOX-GFP) nanosystem that minimises off-target effects by pH-sensitive charge transition, enhancing tumour selectivity, reducing systemic toxicity, and ensuring efficient drug delivery in breast cancer therapy [74]. Yu et al. develop NTZ-DCMMs, charge-reversible nanocarriers with enhanced EGFR-targeted drug delivery, ensuring tumour-specific, redox-triggered DOX release, minimising off-target toxicity, cardiotoxicity, and enabling synergistic chemo-photodynamic therapy for hepatocellular carcinoma [75]. Chen et al. developed a reversible pH-responsive nanocarrier with poly(tannic acid)-coated MSNs that enables controlled drug release, HER2-targeting, and reduced off-target effects, enhancing efficacy while minimising secondary side effects in cancer therapy [76].

#### 3.2 Controlled drug release

Biodegradable nanoparticles and micelles offer significant potential as nanosystems for delivering powerful anticancer drugs directly to target sites. Using specific polymers as nanocarriers allows for targeted drug delivery and controlled release development. Xia et al. developed redox/pH dual-responsive nanoparticles with reversible surface charge which were prepared using HA-SH and CS for the controlled release of the anticancer drug DOX [77]. Zhou et al. developed a pH-responsive charge-reversal polyelectrolyte, and an integrin αVβ3 antibody-functionalized GO complex was developed for targeted, controlled release of DOX, enabling selective drug release in acidic intracellular organelles for enhanced cancer treatment [78]. Wang et al. developed a pH-responsive, charge-reversible, and photo-cross-linkable polymer nanoparticle composed of [poly(VBMC-*co*-AA)] and a block of [poly(DMAEMA-*co*-St)] for controlled DOX release. This nanocarrier enabled pH and light-triggered release adjustments, showed effective tumour cell inhibition in vitro, and potential for precision-controlled drug delivery [79]. Zhang et al. synthesised a pH-responsive, charge-reversal nanocarrier, PAH-cit/APTES-MSNs, which was developed for controlled drug release, effectively delivering doxorubicin to the nucleus of HeLa cells, showing a promising result for targeted cancer therapy [80].

#### 3.3 Combination therapy (or) co-delivery

Single-drug treatments often fall short therapeutically and risk tumour cell resistance, whereas combination therapy uses multiple agents to enhance effects and reduce resistance. CR-NDDSs-based co-delivery systems, including dual-drug, dual-gene, and co-loading systems of drug-and-gene, are also expected to be used in the combined treatment of tumours [88]. Jia et al. reported a dual-responsive shape-transformable charge-reversible nanoparticle (DHP@BPP) combined with chemo-photodynamic immunotherapy for treating breast cancer and lung metastasis [81]. Hu et al. developed a hybrid nanocarrier, UPOE, using stimuli-responsive, charge-reversal metal–organic-framework-based polymer-coated nanoparticles to improve co-delivery of doxorubicin and cisplatin, enhancing combination therapy for multidrug-resistant cancer [31]. The pH-responsive, charge-reversal sericin-based nanocarrier MR-SNC was developed by Aghaz et al. for the co-delivery of resveratrol and melatonin to MCF-7 breast cancer cells, achieved efficient cellular uptake and significant cytotoxicity, which led to cell apoptosis in acidic conditions [82]. Guo et al. designed dual-pH-responsive, CRNs DMMA-P-DOX/LAP for co-delivering doxorubicin and lapatinib to breast cancer cells, enabling effective tumour penetration and notable reduction in MCF-7 tumours, with excellent biosafety in vivo [83].

#### 3.4 Improved cellular uptake

Upon exposure to mildly acidic conditions of the tumour microenvironment (around pH 6.5–6.8), ionizable groups such as amines, imidazoles, or carboxyl-based moieties, CRNs become protonated or lose their protective shells, leading to a shift in surface charge from negative to positive. This transformation enhances electrostatic attraction toward negatively charged tumour cell membranes, thereby facilitating more efficient endocytosis and intracellular delivery of therapeutic agents. [87]. Zhou et al. developed a dual pH-responsive nanocarrier, CRN (PMLA-PEI-DOX-TAT@PEG-DMA), designed for tumour-targeted drug delivery with improved specificity and efficacy. This system leverages distinct pH-triggered mechanisms to enhance drug release within the acidic tumour microenvironment while maintaining stability in physiological conditions, resulting in significantly enhanced anti-tumour activity and reduced systemic toxicity. The dual pH-responsiveness enables precise control over therapeutic delivery, optimizing treatment outcomes [84]. Another straightforward approach to enhance the uptake of nanocarriers (NCs) by tumour cells is by increasing their positive surface charge. However, as previously mentioned, highly positive NCs tend to be cytotoxic. To address this issue, Feng et al. developed a pH-sensitive nanocarrier based on a cisplatin (IV) prodrug-loaded charge-reversal system (CDs-Pt (IV)@PEG-PAH/DMA) for imaging-guided drug delivery, which enhanced cancer therapeutic effects [85].

### 4 Preclinical and clinical insights of charge-reversible nanocarriers

Preclinical studies have revealed that CRNs significantly improve antitumour performance by enhancing tumour accumulation, cellular uptake, and controlled drug release in response to tumour microenvironmental cues. Different CRN architectures, including pH-responsive polymeric micelles, lipid-based nanoparticles, and dendrimer-based systems, have demonstrated improved tumour penetration and minimised systemic toxicity in murine and xenograft models. Additionally, CRNs have shown the ability to overcome multidrug resistance through effective endosomal escape and cytoplasmic delivery of therapeutic agents. While clinical evaluation is still in its preliminary stages, several CRN formulations are being assessed in phase I/II clinical trials for their safety, pharmacokinetic behaviour, and therapeutic potential. Collectively, these findings underscore the promise of CRNs as next-generation, stimulus-responsive drug delivery platforms capable of achieving precise tumour targeting with reduced off-target effects. Li et al. developed a dual immune checkpoint-inhibiting nanocarrier, aLS@VpNPs, which is cloaked with triple-negative breast cancer (TNBC) cell membranes and incorporates anti-LAG3 and Siglec10 proteins. This biomimetic design enhances tumour targeting and biocompatibility. Moreover, the nanocarrier is combined with photodynamic therapy (PDT), where light-triggered ROS generation induces immunogenic cell death, effectively transforming immunologically “cold” tumours into “hot” ones. Consequently, this combination synergistically potentiates the efficacy of dual checkpoint blockade by activating both T cells and macrophages, offering a promising therapeutic strategy for TNBC [89]. In a similar approach, Yang et al. designed biomimetic nanovaccines targeting the CXCR4 receptor, incorporating ROS-responsive cores to simultaneously deliver STING agonists and tumour-associated antigens to dendritic cells through macropinocytosis. This strategy effectively activated the STING pathway within the cytosol, leading to a strong adaptive immune response [90].

Charge-reversible nanocarriers are also advancing the field of gene-based immunotherapy. In a 2024 study, Wang et al. developed MSN@MT nanocarriers composed of dendritic mesoporous silica coated with manganese ions and tannic acid, specifically designed to activate the cGAS-STING pathway in dendritic cells. This approach significantly improved antigen cross-presentation and stimulated T-cell activation in murine models, resulting in strong anti-tumour responses [91]. In addition to vaccines, CRNs are now being applied to deliver gene-editing systems such as CRISPR-Cas9. Nie et al. emphasised the advancement of stimuli-responsive nanoplatforms such as pH-sensitive structures and redox-responsive polymers that enable protected and precise intracellular transport of CRISPR components [92]. Ben-Akiva et al. engineered biodegradable, lipophilic polymer-based nanocarriers capable of systemically delivering mRNA along with TLR agonists to dendritic cells in the spleen, leading to strong activation of CD8^+^ T cells and effective tumour suppression in mouse models [93]. Most CRNs are still at the preclinical level; however, notable strides have been made toward their clinical implementation. While CRN-specific platforms have yet to appear in phase I/II clinical trials, early stage studies involving pH-sensitive and ionizable lipid-based nanoparticles for mRNA and gene-editing delivery are underway. Liang et al. highlight that these emerging technologies lay the groundwork for CRNs to become key components in future personalized cancer therapy approaches [32].

### 5 Challenges and limitations

CRNs offer a significant advantage over traditional nanocarriers by promoting higher cellular uptake and minimizing nonspecific interactions, thereby enhancing therapeutic accuracy. However, despite their potential, several challenges limit their clinical translation. Regulatory authorities, including the FDA and EMA, require extensive safety assessments due to the complex stimuli-responsive nature of CRNs, which can affect immunogenicity, cytotoxicity, and long-term biocompatibility [94]. Research showing low toxicity at high concentrations for specific nanocarriers emphasizes their advantages over inorganic substitutes, such as silica and gold nanoparticles, which are often associated with safety concerns [95]. Despite these encouraging findings, comprehensive evaluation and mitigation of safety issues remain essential to facilitate the successful translation of nanocarrier technologies from experimental research to clinical application. Addressing challenges such as long-term biocompatibility, accumulation, and dose-dependent toxicity will be critical for advancing these promising systems towards safe and effective patient use [96].

Their intricate structural design, composed of multiple functional elements, complicates standardization and reproducibility among various formulations [97]. In addition, the synthesis of CRNs often involves elaborate multistep processes to achieve accurate charge-switching properties, which can lead to inconsistencies between production batches and create scalability challenges [98]. The inclusion of diverse functional groups and the need for precise control over physicochemical characteristics further increase manufacturing costs and delay clinical development. Maintaining stability during storage is another critical concern, as environmental factors such as temperature fluctuations and light exposure may induce premature charge reversal, thereby altering drug release profiles and reducing therapeutic efficacy [99].

The clinical translation of CRNs remains challenging due to interpatient variability, tumour microenvironment heterogeneity, and differential immune responses, all of which significantly impact their pharmacokinetics, biodistribution, and therapeutic efficacy [100]. These factors collectively influence the biological behaviour and therapeutic efficacy of CRNs, complicating their predictability and consistency in patients. In addition, the intricate composition of these systems often extends regulatory approval and clinical evaluation processes. Ethical and environmental considerations further complicate their translation, as the degradation of by-products and long-term accumulation of synthetic nanomaterials in ecosystems necessitate thorough investigation [101]. To address these limitations, current research focuses on multiple strategies, including standardizing large-scale production to ensure consistency, developing formulations with superior physicochemical stability, and adopting personalized medicine approaches to optimize CRN efficacy based on individual patient profiles [102]. Furthermore, comprehensive environmental impact assessments are vital to promote safe, ethical, and sustainable applications of CRNs in clinical practice.

### 6 Future perspectives

Future development of charge-reversible nanocarriers must prioritize translating proof-of-concept systems into clinically viable drug delivery platforms by emphasizing biodegradability, biocompatibility, and high positive surface potentials to optimize cellular uptake and endosomal escape. Addressing safety and regulatory challenges, especially for inorganic materials such as carbon nanotubes and mesoporous silica nanoparticles and optimizing polymeric nanocarriers such as PAMAM dendrimers are critical for minimising toxicity and enhancing clinical applicability. Advancing FDA-approved excipients with enzymatically cleavable coatings, innovating adaptable charge-reversal mechanisms responsive to heterogeneous tumour microenvironments, and exploring alternative activation modalities beyond photoresponsive systems will accelerate clinical translation and broaden applications beyond oncology to include retinal therapy, inflammatory diseases, and mRNA vaccine delivery, heralding a new era of personalized, multimodal, and highly effective therapies driven by the next generation of charge-reversible nanocarriers.

## Conclusion

CRNs enable targeted cancer therapy by maintaining neutral or negative charge in circulation and switching to positive charge in the acidic tumour microenvironment, enhancing cellular uptake and therapeutic efficacy. This switchable charge feature boosts therapeutic effectiveness and minimises harm to healthy tissues, positioning these systems as promising candidates for clinical application. The adaptability of CRNs is characterised by their ability to respond to a broad spectrum of stimuli, including changes in pH, enzymatic activity, redox environments, temperature, light, ultrasound, X-rays, and magnetic fields. These stimulus-responsive mechanisms enable precise, site-specific drug release, significantly improving the accuracy of cancer therapies. For example, pH-sensitive nanocarriers utilise the acidic conditions of the tumour microenvironment to trigger charge switching, enzyme-responsive systems to activate in the presence of tumour-associated enzymes, and redox-sensitive carriers to release drugs in response to elevated intracellular glutathione levels.

Furthermore, advanced platforms employing external stimuli such as light, ultrasound, or magnetic fields offer the advantage of precise spatiotemporal control over therapeutic delivery. These approaches have shown promising outcomes in preclinical studies, supporting the development of combination treatments and co-delivery systems applications that integrate therapeutic and diagnostic functions. While challenges such as stability, large-scale production, and regulatory approval remain, ongoing progress in nanocarrier engineering and materials innovation continues to drive this field forward. Ultimately, CRNs can potentially transform cancer therapy by offering safer, more efficient, and highly individualised treatment options.

## Data Availability

Data sharing is not applicable as no new data was generated or analyzed in this study.
